# Clinico-epidemiological study of spinal injuries in a predominantly rural population of eastern Nepal: A 10 years' analysis

**DOI:** 10.4103/0019-5413.36988

**Published:** 2007

**Authors:** Suraj Bajracharya, Mahipal Singh, Girish Kumar Singh, Bikram Prasad Shrestha

**Affiliations:** Department of Orthopaedics, B. P. Koirala Institute of Health Sciences, Dharan, Nepal

**Keywords:** Clinico-epidemiological study, retrospective review, spinal injuries, treatment of spinal injuries

## Abstract

**Background:**

A clinico-epidemiological study helps to plan future preventive measures and management strategies for spinal trauma. This is a 10 years' retrospective review of spinal-injury patients treated at a tertiary health center in the eastern of Nepal to determine clinico-epidemiological aspects of spinal-injury patients in a predominantly rural population of eastern Nepal.

**Materials and Methods:**

All medical record files of patients with spinal injury from 1996 to 2005 in the Medical Record Section of BPKIHS (B. P. Koirala Institute of Health Sciences) were studied. The preformed pro forma consisting age, sex, place of living, mode of injury, hospital stay level of injury, site of injury, associated injury, Frankel grading of neural deficit and treatment modality was filled from the record files of patients. These parameters were entered in Excel 8 and analyzed by EPI INFO 2002. Details of 896 patients of spinal injury were recorded in the 10-year period of review.

**Results:**

684 (76.35%) male and 212 (23.66%) female patients with mean age of 41.74 ± 16.53 years and 38.56 ± 15.86 years respectively were studied. Two hundred forty-two (27%) patients were from hilly districts of eastern Nepal. Fall from height [in 350 (39%) patients] was the commonest mode of spinal injury. Six hundred thirty-six (71%) patients presented with a neurological deficit. Seven hundred thirty-three (85%) patients were treated conservatively, compared to 163 (15%) surgically treated patients. One hundred forty-six (22%) patients were treated with operative interventions in the last five years.

**Conclusion:**

The study shows that the most vulnerable group for spine injury was the group of patients of productive age with late presentation (i.e., injury hospital duration − 41.64 ± 54.24 hours) without proper prehospital management. The treatment modalities have changed (from conservative to surgical) in this part of the world. These specific observations help us in further planning for preventive measures and management in our setting.

The skeletal injuries and head injuries are the most common injuries sustained by trauma patients. Skeletal injuries occur in 78% of multiply injured patients.^1^ Spine-injury prevalence in trauma patients is 6%. Out of all the spinal fractures, 90% occur between T-11 and L-4 vertebra.^1^ Spinal injury frequently involves multiple, noncontiguous vertebral levels. Multilevel spine fractures are present in 15-20% of spine-injury patients.^2^ Traumatic spinal cord injury occurs most frequently in the cervical region (50-64%).^3^ Lumbar injuries represent 20-24% of traumatic spinal cord injuries; and thoracic injuries, 17-19%. Half of the patients admitted for spinal cord injury have a complete neurologic injury on initial examination, with no preservation of motor or sensory function below the injury level. Tetraplegia is more often incomplete than complete. Paraplegia is more often complete. Concomitant major injuries to other regions occur in 37% of patients with a thoracolumbar fracture.^4^

The exact data about the spine-injured patients cannot be found in this part of the world. Unfortunately, ours is a country which does not have uniform surgical facilities. Patients have to travel a long distance to get treatment without the availability of proper and quick transportation. Most people do not have medical insurance to cover the cost of treatment. Hence it was considered worthwhile to have a retrospective hospital-based analysis of such type of injuries in order to know the actual burden and its consequences so that further planning in terms of preventive measures, as well as management protocol, can be made accordingly.

## MATERIALS AND METHODS

This study is a retrospective hospital-based analysis of spine-injured patients that presented to the Department of Orthopedics, B. P. Koirala Institute of Health Sciences (BPKIHS), Dharan, Nepal. The study included all patients that attended the BPKIHS from April 1996 (Baishak 2053) to May 2006 (Chaitra 2062) with spinal injury of all levels (cervical, thoracic, lumbar, sacro-coccygeal), by searching all medical record files from the Medical Record Section. Clinico-epidemiological aspects of patients – mainly, age, sex, place of living, mode of injury, hospital stay duration, level of injury, site of injury, associated injury, Frankel grading of neurological deficit and treatment modalities – were recorded in a preformed pro forma. Data was entered in Excel 8 sheet and was analyzed with the help of EPI INFO 2002 computer-based statistical analysis for frequency, mean, tables and correlation. The data is presented in tables as well as bar and pie chart forms.

## RESULTS

**Figure 1:**
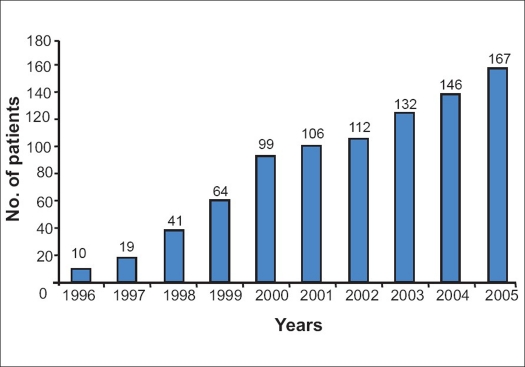
Showing increasing pattern of incidence of spine-injured patients at BPKIHS for the last 10 years

Details of 896 patients of spinal injury in the 10-year period were recorded. There was obvious incremental pattern in incidence of spinal injury attended to at BPKIHS [Figure 1]. There were only 10 patients of spinal injury recorded in the year 1996 (2052/2053), which increased to 167 in 2005 (2061/2062).

There were 684 (76.35%) male and 212 (23.66%) female patients with mean age of 41.74 ± 16.53 years and 38.56 ± 15.86 years respectively [Tables 1A and 1B]. Three hundred fifty (39.1%) injured patients were in the productive age groups ranging from 21 to 41 years. The spine-injured patients reported from hilly districts of eastern Nepal – like from Sunsari, 188 (21%); Dhankuta, 161 (18%); Morang, 134 (15%); Bhojpur, 81 (9%); compared to Terai districts of eastern Nepal – like Jhapa, 63 (7%); Siraha, 98 (11%); Saptari, 108 (12%); others, 63 (7%) [Table 2].

**Table 1A:** Age and sex of patients

	Number of patients	Mean age (years)
Male	684 (76.35%)	41.74±16.53
Female	212 (23.66%)	38.56±15.86

**Table 1B:** Age distribution table

Range of age (years)	No. of patients no. of	Cumulative no. of patients
< 20	110	110
21-30	184	294
31-40	166	460
41-50	190	650
> 51	246	896

**Table 2:** Location of patients

District	Geography	No. of patients	Percentage
Sunsari	Foot hill	188	21
Dhankuta	Hilly	161	18
Morang	Terai	134	15
Bhojpur	Hilly	81	9
Jhapa	Terai	63	7
Siraha	Terai	98	11
Saptari	Terai	108	12
Others	Mixed	63	7

Fall from height [in 350 (37.86%) patients] was the commonest mode of spinal injury, followed by fall from tree in 188 (21%), road traffic accident in 116 (13%), farm-related injuries in 72 (8%) and other modes in 170 (19%) patients. Fall from trees while cutting leaves, fall from unbarricaded first/ second floor verandah, fall while trying to repair roofs, slip from hill slope, slip while carrying weight on head were notable modes of injury reported. Only 135 (15%) patients got primary prehospital management from the referral center at the district hospitals. Injury hospital duration was 41.64 ± 54.24 hours, and hospital intervention duration was 2.023 ± 0.606 hours. Colles' fracture, calcaneal fractures, pelvic fractures were commonly associated and were only present in 150 (17%) patients out of the total cases.

Cervical spine injuries 358 (40%) were the most common spine injuries, followed by lumbar, thoracic and sacral injuries. 302 (34%) out of all the patients had Frankel grade A; and 239 (27%) patients had Frankel grade E. The correlation between the neurological status by Frankel grading and the level of injury at the time of presentation is shown in [Table 3].

**Table 3:** Correlation between ASIA scoring and level of spine injury

ASIA scoring	Total number of patients	Level of spine injury				
		
		Cervical	Thoracic	Lumbar	Sacral	SCIWORA
A	302 (34)	109	68	112	5	8
B	52 (5)	22	7	20	1	2
C	164 (18)	54	39	63	1	7
D	139 (16)	63	12	58	2	4
E	239 (27)	110	45	78	0	6
		358 (40)	171 (19)	331 (37)	9 (1)	27 (3)

Figure parentheses indicate percentage

Two hundred sixteen (93%) out of 233 patients were treated conservatively, compared to operative treatment in 17 (7%) patients in the initial five years of the study period; in comparison, 146 (22%) out of 663 patients were operated in the last five years. Modalities used for spinal injuries are shown in [Table 4].

**Table 4:** Treatment modalities in the initial five years and the last five years at BPKIHS

Treatment modalities	Initial 5 years	Last 5 years	Total
A. Conservative	216 (93)	517 (78)	733
B. Operative treatment modalities	17 (7)	146 (22)	163
1. Hartshill Fixation with decompression and CBG	3	32	35
2. Hartshill Fixation and CBG	8	64	72
3. Pedicle screw with Decompression and CBG	2	14	16
4. Pedicle screw and CBG	3	32	35
5. Posterior cervical spine triple wiring	1	2	3
6. Anterior and posterior fixation of cervical spine with CBG	0	2	2

Figure parentheses indicate percentage

## DISCUSSION

Retrospective studies are usually guideline studies for any specific area, region or country to know about the exact statistics regarding the events or diseases at that specific place. This is a large retrospective study of spine-injured patients in the last 10 years in a predominantly rural population in the eastern region of Nepal. It gives an overview of clinico-epidemiological characteristics and burden of spine-injured patients, the delay in presentation of such injuries and their prehospital management. This study will formulate a basis to evaluate the treatment modalities offered to these patients and help to plan preventive aspects of such injuries at the community level.

These patients took mean 41.64 ± 54.24 hours to reach the hospital, as they traveled long distances from the site of injury, or the delay was caused by the referral center located near their location. We have reviewed some of the large retrospective studies of spine and spine-related injuries at different places and in different situations, which are similar to our study but of different importance and with valued recommendations and suggestions.

Pickett *et al.*^5^ reviewed and described the incidence, clinical features and treatment of traumatic spinal cord injury (SCI) treated at a Canadian tertiary care center, considered essential for public resource allocation and primary prevention. They retrospectively reviewed hospital records of all patients (n = 151) with traumatic SCI between January 1997 and June 2001. Variables assessed included age, gender, length of hospitalization, type and mechanism of injury, associated spinal fractures, neurologic deficit and treatment. Annual age-adjusted incidence rates were 42.4 per million for adults aged 15-64 years and 51.4 per million for those 65 years and older. Motor vehicle accidents accounted for 35% of SCIs. Falls were responsible for 63% of SCIs among patients older than 65 years and for 31% of injuries overall. Cervical SCI was the most common, particularly in the elderly, and was associated with fracture in only 56% of cases. Thoracic and lumbar SCIs were associated with spinal fractures in 100% and 85% of cases respectively. In-hospital mortality was 8%. Mortality was significantly higher among the elderly. Treatment of thoracic and lumbar fractures associated with SCI was predominantly surgical, whereas cervical fractures were equally likely to be treated with external immobilization alone or with surgery. They concluded that a large proportion of injuries were seen among older adults, predominantly as a result of falls. They recommended prevention programs should expand their focus to include home safety and avoidance of falls in the elderly.

Sommer *et al.*^6^ reported epidemiology, treatment, clinical and radiological results of 283 patients with spine fractures in a five-year period. The operation rate ranged from 42% of cervical to 9% of thoracic and 24% of the lumbar spine. He found good radiological results concerning the correction of the wedge compression and the collapse of the lumbar vertebral body by fixation with an internal fixator. After a followup of 2-5 years, nearly 80% of conservatively, as well as surgically, treated patients had residual back pain. A correlation between the intensity of pain and the radiological dislocation, initial diagnosis with instability was found, hence adequate conservative or operative treatment is very important and outcome was similar in conservative and operative treatment.

Our study is a hospital-based review, mainly concentrating on creating awareness about the fact that spinal injury is alarmingly increasing in this part of the world and we should timely think of preventive aspects. As we have seen, we tend to opt for operative modalities and there was a paradigm shift towards operative treatment in our study, We also need to evaluate the long term outcome of treatment (conservative or surgical) in these patients.

Velmahos *et al.*^7^ attempted to characterize spinal injury after falls from height and identify predictive factors of spinal injuries. Medical records of patients with falls from height >10 feet admitted in a Level I trauma center during a period of 66 months were reviewed. Univariate and multivariate analyses were performed to identify independent risk factors of spinal injuries. Of the 414 patients, 127 (31%) suffered 277 spinal injuries. Multiple spinal injuries at different levels were found in 62 (49%) patients; in 19 (15%) patients, spinal injuries were at noncontinuous levels. The only independent predictor of spinal injury was alcohol intoxication (odds ratio = 3.305; 95% CI, 1.75-6.242; *P* < 0.001), but the number of intoxicated patients was low and the predictive ability weak. Level of falls from height did not correlate with likelihood of spinal injury. Because of the absence of reliable predictors of spinal injury, the possibility of multiple noncontinuous fractures and the presence of distracting injuries clouding the clinical presentation, Velmahos GC *et al.* concluded that aggressive evaluation of the entire spine is warranted.^7^

The fall from height was the commonest cause of spinal injury followed by fall from trees in our study. This might be because of the geographic pattern of our region and the need to climb trees for fodder. Injury due to the later can be prevented by awareness, which will certainly decrease the incidence of such dreadful injury in the community.

Though it is only a hospital-based retrospective review, it would certainly prove to be an important document or research work for this subcontinent to enable us to know about clinico-epidemiological characteristics of spine-injured patients in predominately rural population present in our region. The data collection for this study was the major limiting factor in this form of study. As computer-based record-keeping is not available, each and every parameter was searched to the greatest extent from the record files so that commonly available parameters could be studied for the review. However, we could evaluate only few important parameters related to spine injury, even then it gives us valuable information.

## CONCLUSION

The spinal injuries are found to have a rising trend in our setting in a physically active and productive age groups (21-40 years), with resultant paraplegia/ quadriplegia leading to personal and family tragedies being affected and with decline in qualitative and quantitative aspects of life along with socioeconomic burden to the nation. Such injuries should be prevented with a very sound preventive program in the community.

The awareness about primary and secondary prevention among both the target population (mainly productive age groups) and those involved in preventive measures of such injuries. Hence we recommend building barricaded walls or fences in houses; not to climb trees to collect fodder, or if climbing to heights is unavoidable, doing so taking necessary precautionary measures.

There is an urgent need to reduce the delay in bringing the spinal trauma patient from site of injury to tertiary care hospital. This study clearly reflects the increasing burden of patients with spine injury for treatment in this part of the world. There is a need of performing a prospective study with good followup and with comparison between operative and conservative treatment of spinal injuries in the context of direct and indirect costs incurred, neurological recovery and patient's outcome with treatment modalities.
